# The association between peripheral nerve blocks and postoperative delirium in adults undergoing hip fracture surgery: a systematic review and meta-analysis

**DOI:** 10.1186/s42836-025-00346-7

**Published:** 2025-12-22

**Authors:** Bethany Davey, Abdul-Hadi Kafagi, Abdullah Bin Sahl, Anand Pillai

**Affiliations:** 1 Faculty of Biology, Medicine and Health, The University of Manchester, Manchester, M13 9PL UK; 2 Department of Trauma & Orthopaedics, Wythenshawe Hospital, Manchester University Hospitals NHS Foundation Trust, Manchester, M23 9LT UK

**Keywords:** Postoperative delirium, Hip fracture, Peripheral nerve block

## Abstract

**Objective:**

To review and synthesise the existing evidence on the effects of peripheral nerve block (PNB) compared with no nerve block on the incidence of postoperative delirium (POD) in adults undergoing hip fracture repair.

**Methods:**

A systematic search of electronic databases (PubMed, Web of Science, EMBASE, and the Cochrane Library) for relevant literature published from database inception to 1st May 2025 was conducted. Randomised controlled trials with PNBs as an intervention in adults undergoing surgery for hip fractures were selected. Studies that excluded patients with preoperative cognitive dysfunction and observed POD as an outcome were eligible.

**Results:**

Twelve randomised controlled trials, involving a total of 1157 participants, 602 in the control group and 555 in the intervention, were included for quantitative analysis. The meta-analysis revealed that PNBs significantly reduced the incidence of POD (RR: 0.68, 95% CI [0.50 to 0.91], *P* = 0.009, I^2^ = 43%).

**Conclusion:**

In patients without pre-existing cognitive impairment, the perioperative use of PNBs can reduce the occurrence of POD. However, substantial variation in study design and heterogeneity of PNB approaches limit the certainty of these findings. Future research calls for well-designed, standardised, and stratified clinical trials to compare the efficacy of each PNB approach and to evaluate their potential benefits in those at greater risk of POD, including those with baseline cognitive impairment and preoperative delirium.

Video Abstract

**Supplementary Information:**

The online version contains supplementary material available at 10.1186/s42836-025-00346-7.

## Introduction

Hip fractures are a major cause of morbidity and mortality in older adults, representing approximately 95% of total fractures in individuals over 60 years [1–3]. With global ageing, the incidence is projected to rise to 6.3 million annually by 2050—a 300% increase since 2000 [4]. In England, one in 45 hospital beds is occupied by a hip fracture patient [5]. Management requires multidisciplinary care, with postoperative goals centred on effective pain control, early mobilisation, and prevention of complications.

Postoperative delirium (POD) is among the most frequent and consequential complications following hip fracture surgery, affecting up to 50% of patients [6–8]. Its pathophysiology is multifactorial, involving the interplay of trauma, surgical stress, anaesthesia, and neuroinflammation [9, 10]. POD is associated with longer hospital stays, functional decline, increased mortality, and greater risk of institutionalisation and readmission. [16–21] In particular, mortality may be up to six times higher among patients who develop POD. [20].

Both non-modifiable and modifiable factors contribute to POD risk. Older age, male sex, frailty, and comorbidities such as atrial fibrillation and chronic obstructive pulmonary disease are established predictors. [19, 22, 23] Modifiable perioperative factors, such as malnutrition, poor glycaemic control, surgical delay, intraoperative blood loss, and prolonged operative time, also play significant roles. [19, 24–28] Addressing these factors through multidisciplinary optimisation is therefore a key strategy for prevention.

Pain is a critical and modifiable risk factor for delirium [29]. Inadequate analgesia can precipitate acute cognitive dysfunction, while excessive opioid use increases the risk of delirium and respiratory depression [30–32]. Optimal pain control in hip fracture patients is essential not only for comfort but also to facilitate positioning, anaesthesia, and early mobilisation—key determinants of recovery and discharge [33, 34].

Analgesic strategies include systemic agents (paracetamol, NSAIDs, opioids) and regional techniques such as peripheral nerve blocks (PNBs). Commonly used PNBs for femoral neck fractures include the femoral nerve, fascia iliaca, psoas compartment, and pericapsular nerve group (PENG) blocks [35, 36]. These techniques improve pain scores, facilitate mobilisation, and reduce opioid requirements [37–39]. Consequently, PNBs may reduce the incidence of POD through opioid-sparing effects and improved analgesia. However, current evidence is inconsistent. The 2020 Cochrane review by Guay et al. found limited and heterogeneous data regarding the relationship between PNBs and delirium, and several randomised trials have since been published [40].

This systematic review and meta-analysis aims to synthesise the latest evidence on the association between peripheral nerve blocks and postoperative delirium in adults undergoing hip fracture surgery. By updating and expanding upon prior work, it seeks to clarify whether PNBs confer a protective effect against POD and inform perioperative practice.

## Methods

The Preferred Reporting Items for Systematic Reviews and Meta-Analyses (PRISMA) guidelines constitute the framework for this systematic review and meta-analysis [41].

### Objectives

To compare the effects of the use of PNBs as perioperative analgesia versus no nerve block on the outcome of POD in adults with hip fractures.

### Literature search and study selection

A research question was defined via the PICO framework. The population included patients over the age of 18 with hip fractures of any kind. The intervention consisted of PNBs of any kind administered perioperatively. The comparison was standard care with no nerve block or a placebo. The outcome of interest was POD.

Four electronic databases (PubMed, EMBASE, Web of Science, and the Cochrane Library) were searched by two independent authors (BD and AHK) for relevant published articles from inception to 5th May 2025. Reference lists of secondary research found to be relevant were also screened.

The search terms for PubMed were as follows: (((postoperative[MeSH Terms]) OR (postsurgical) OR (perioperative) OR (post repair))) AND ((delirium[MeSH Terms]) OR (confusion) OR (cognitive dysfunction) OR (acute confusion) OR cognitive impairment)) AND ((neck of femur fracture) OR (femoral neck fracture) OR (hip fracture[MeSH terms]) OR (proximal femur fracture) OR (subcapital fracture) or (intertrochanteric fracture) OR (subtrochanteric fracture) OR (trochanteric fracture) OR (hip arthroplasty)) AND ((peripheral nerve block[MeSH Terms]) OR (PNB) OR (femoral nerve block) or (fascia iliaca block))). This search was adapted to suit the format of the remaining three databases.

Initial screening for potentially relevant studies was conducted. First, papers were reviewed by title and abstract by two authors (BD and AHK). Studies observing the effect of any type of PNB on outcomes in hip fracture patients were collected. Full text screening according to the inclusion and exclusion criteria was then carried out. In cases where the full text was not available, authors were contacted via email. Literature that met the eligibility criteria was included in the meta-analysis. Instances of disagreement in the selection process between the authors were resolved through discussion and consensus.

The inclusion criteria were as follows: (1) parallel-group randomised control trials; (2) population comprised of adults over 18 admitted to hospital with hip fracture; (3) POD observed as an outcome; (4) PNB used as the intervention.

Studies were excluded if: (1) full text or results were unavailable; (2) English translations were unavailable; (3) research was not primary (e.g., reviews, protocols, or editorials); (4) comparator did not include a control group without nerve blocks, or (5) patients with pre-operative delirium were included in the population.

### Data extraction

The following were extracted from each study: study design; population size; setting and duration; mean age and standard deviation; inclusion and exclusion criteria; type of PNB; time of PNB placement; surgical anaesthetic used; PNB technique, drug, and dose; total POD incidence; POD diagnostic tool and time periods. The information was stored in an Excel spreadsheet. Two authors cooperatively collected and documented the data. Any discrepancies were resolved through discussion among the authors to ensure consistency and accuracy.

### Outcome measures

The incidence of POD was the primary outcome. If delirium incidence was reported at multiple time points, to avoid overcounting, delirium incidence at 48 h postoperative was used.

### Data synthesis

The pooled log risk ratios (logRR) and corresponding 95% confidence intervals (CI) were calculated for the dichotomous data of each study. The Mantel–Haenszel method and fixed-effect model were used for the analysis. To account for variance within each study, individual study weights were calculated by the inverse variance method. For a visual summary of the logRR estimates, CIs, and the overall pooled effect, forest plots were generated. For each plot, the area of the circle is in proportion to the weight of the estimate. *P*-values < 0.05 were considered statistically significant for overall effect estimation. The methods described above were carried out in RevMan Web.

### Unit of analysis issues

If a study involved two intervention groups, the control group was split evenly for comparison. If studies divided delirium into severity subgroups, this data was combined for a total delirium incidence. In cases of two control groups, for example, spinal anaesthesia and general anaesthesia, the two groups were combined.

### Assessment of heterogeneity

The degree of heterogeneity among the studies was evaluated with Cochrane’s Q test (X^2^). The I^2^ statistic was used to quantify the level of inconsistency. This was interpreted as: low heterogeneity (0%–25%), moderate heterogeneity (25%–75%), or high heterogeneity (75%–100%).

### Assessment of risk of bias

The risk of bias for the outcome of POD regarding the effect of assignment to intervention was assessed using Cochrane ROB 2. The evaluation involved five categories, with the risk of bias arising from: the randomization process, deviations from the intended interventions, missing outcome data, measurement of the outcome, and selection of the reported result. After the signalling questions were answered, each domain was scored as low risk, some concerns, or high risk. These components amalgamate to produce an overall risk of bias for each trial.

### Assessment of the certainty of the evidence

For the outcome of POD, the GRADE approach was used to evaluate the certainty of evidence. The criteria for downgrading in each domain are described in Table 1.

**Table 1:** Summary of the criteria for downgrading certainty of evidence using the GRADE tool

Domain	Criteria for Downgrading Certainty of Evidence
Risk of Bias	Certainty was downgraded by one level if most of the included trials were assessed as having some concerns regarding risk of bias. If exclusion of studies at high risk of bias resulted in a meaningful change in the overall conclusion, certainty was downgraded by two levels
Heterogeneity	Certainty was downgraded by one level if the I^2^ statistic exceeded 50%, and by two levels if it exceeded 75%
Indirectness	Certainty was downgraded by one level when substantial differences were identified in the intervention techniques, outcome measures, comparator groups, or populations across the included studies
Imprecision	Certainty was downgraded if the confidence interval for the pooled risk ratio included both no effect and a clinically meaningful effect. Additionally, certainty was downgraded when the total sample size across studies was fewer than 400 participants
Publication Bias	Certainty was downgraded by one level if evidence of publication bias was detected based on asymmetry in a contour-enhanced funnel plot or a significant Egger’s test

## Results

### Results of the search

**Fig. 1:**
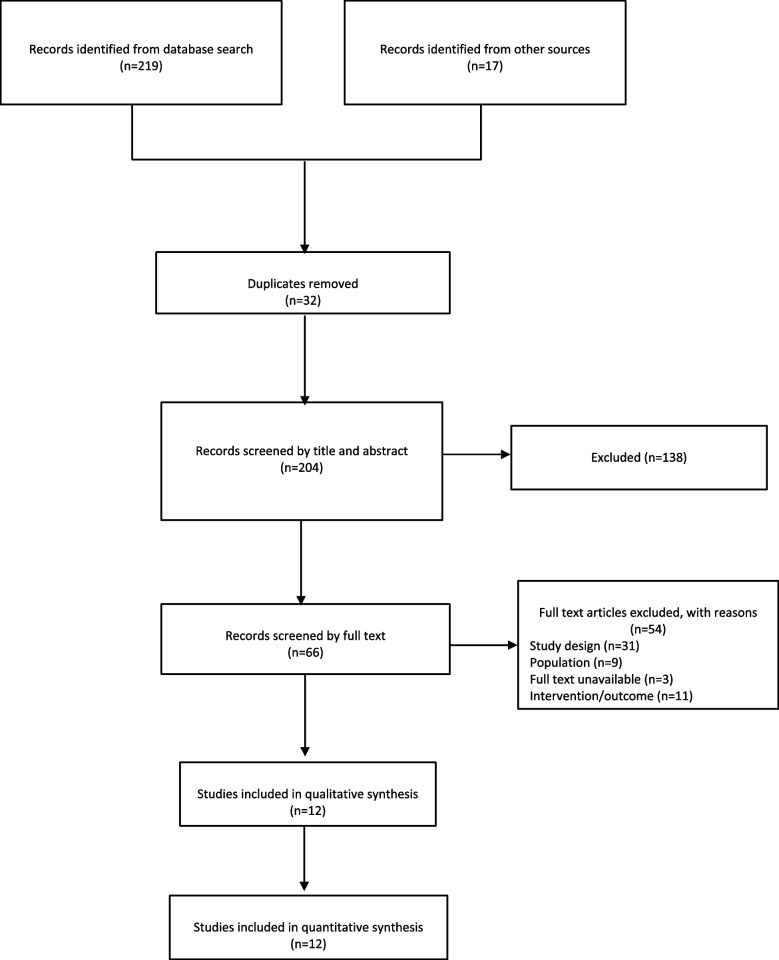
PRISMA flow diagram of study selection

The search strategy retrieved 204 articles. After thorough screening, 12 eligible studies were used for quantitative and qualitative analysis. The screening process is described in Fig. 1.

### Baseline characteristics

Table 2 displays the characteristics of the included trials. Among the 12 studies, seven administered the PNB solely preoperatively [42–48], four used PNB perioperatively [49–52] and one gave a PNB postoperatively [53]. Five studies observed a fascia iliaca compartment block as the PNB [42, 43, 48, 50, 53]. Lumbosacral plexus block was performed in two studies [44, 46] and another two studies used femoral nerve blocks alone [45, 52]. The remaining three studies used a combination of nerve blocks: a psoas compartment block and sciatic nerve block [49]; a fascia iliaca compartment block and femoral nerve block [51]; a lateral femoral cutaneous block and a pericapsular nerve group (PENG) block [47]. Four studies administered the PNB as a single bolus [43, 44, 46, 47], two studies used repeated bolus [45, 50] and six studies gave blockade by bolus and catheter infusions [42, 48, 49, 51–53]. The details of the comparators and supplementary analgesia used in each study are described in Table 2. Further demographics of each study are presented in Table 3.

**Table 2:** Summary of the characteristics of the eligible studies, detailing interventions, comparators, and outcome measures

Study	Time of block placement	Surgical anaesthesia	Block(s)	Block drug and dose	Block technique	Comparison	Supplemental analgesia for both groups	Delirium assessment	
								**Tool**	**Timing**
Nie 2015 [53]	Post-operative—after closure of the surgical wound	General anaesthesia	Fascia iliaca compartment block	Bolus—20 mL, 25 mL or 30 mL 0.5% ropivacaine Infusion pump—0.1 mL/kg/h 0.25% ropivacaine, removed 48 h post-surgery	Landmarks	No block IV PCA with fentanyl and tropisetron	Oral Acetaminophen Oral Dihydrocodeine-tartrate Intramuscular morphine	CAM	Daily screening
Hao 2019 [42]	Pre-operative—on arrival at the emergency department	Epidural anaesthesia	Fascia iliaca compartment block	Bolus—30 mL 0.45% ropivacaine Infusion pump—6 mL/h 0.25% ropivacaine, total 200 ml, approx 33 h	Ultrasound guidance	Placebo—pump with 0.9% sodium chloride	Intramuscular fentanyl 0.05 mg Patient controlled epidural analgesia	CAM	1 h post-surgery then bidaily
Bielka 2021 [49]	Pre-operative intraoperative Postoperative	1. Sedation with block 2. Spinal anaesthesia 3. General anaesthesia	Psoas compartment block AND	Pre-operative Infusion pump—6–8 mL/h 0.125% bupivacaine, timing not specified Post-operative Infusion pump—6–8 mL/h 0.125% bupivacaine, timing not specified	Ultrasound guidance	No block	Oral paracetamol 3 g/day Oral Dexketoprofen 75 mg/day Nalbuphine 5 mg subcutaneous	CAM	__
			Sciatic nerve block	Intraoperative Bolus—450 mg 1.5% lidocaine	Neuro- stimulator				
Yamamoto 2019 [43]	Pre-operative —before spinal anaesthesia	Spinal anaesthesia	Fascia iliaca compartment block	Bolus—40 mL 0.25% levobupivacaine	Ultrasound guidance	No block Post-operative IV acetaminophen	Oral loxoprofen sodium Diclofenac sodium suppository	CAM	__
Tang 2021 [44]	Pre-operative	1. Spinal anaesthesia and sedation 2. General anaesthesia with block	Lumbar plexus block AND	Bolus—20 mL 0.25% ropivacaine	Neurostimulator and ultrasound guidance	No block	IV- PCA with sufentanil and flurbiprofen axetil	CAM	Bidaily for 7 days postoperative
			Sacral plexus block	Bolus—20 mL 0.25% ropivacaine					
Mouzopoulos 2009 [50]	Pre-operative and post-operative	Epidural anaesthesia	Fascia iliaca compartment block	Pre-operative: Bolus—0.25 mg 0.3 mL/kg bupivacaine, every 24 h Post-operative: Bolus—0.25 mg 0.3 mL/kg bupivacaine, every 24 h	Landmarks	Placebo—injection with water	IV paracetamol Intramuscular pethidine	MMSE then DSM-IV and CAM	Daily
Morrison 2016 [51]	Pre-operative —in the emergency department Intraoperative Post-operative	Regional or general anaesthesia	Femoral nerve block AND	Pre-operative: Bolus—20 mL of 0.5% bupivacaine	Ultrasound guidance	No block	Opioids	CAM	Daily
			Fascia iliaca compartment block	Intraoperative: Bolus—15 mL 0.2% ropivacaine up to 24 h later Infusion pump—5 mL/h 0.2% ropivacaine, removed 72 h post surgery					
Uysal 2020 [45]	Pre-operative—in the emergency department	Spinal anaesthesia	Intermittent femoral nerve block	Bolus—0.5 mg/kg 0.25% bupivacaine, every 8 h	Ultrasound guidance	No block IV paracetamol	Epidural catheter, bupivacaine, fentanyl	Delirium rating scale -R—98	For 3 days post operative
Cui 2024 [46]	Pre-operative	General anaesthesia	Lumbar plexus block AND	Bolus—25–35 mL 0.25% ropivacaine	Ultrasound guidance	No block	__	CAM	Daily for 3 days post-operative
			Sacral plexus block	Bolus—20 mL 0.25% ropivacaine					
Yoo 2024 [47]	Pre-operative	General anaesthesia	Pericapsular nerve group (PENG) block AND	Bolus—20 mL 0.375% ropivacaine	Ultrasound guidance	No block	Local infiltration analgesia (ropivacaine, epinephrine, ketorolac, morphine) IV sugamadex IV-PCA fentanyl and ramosetron	CAM	On 2nd day postoperative only
			Lateral femoral cutaneous block	Bolus—5 mL 0.375% ropivacaine					
Loessin 2019 [48]	Pre-operative	__	Fascia iliaca compartment block	1 st Bolus—40 mL 0.124% ropivacaine Infusion pump—10 mL/h 0.2% ropivacaine, until time of surgery 2md bolus—40 mL 0.124% ropivacaine	__	No block	Standard analgesia (eg, opioids, NSAIDs, acetaminophen)	__	__
Rowlands 2018 [52]	Pre-operative—emergency department Intraoperative and Postoperative	Spinal or general anaesthesia	Femoral nerve block	Pre-operative: Bolus—0.5 mL/kg 0.25% levobupivacaine Perioperative: Infusion pump: 5 mL/h 0.2% ropivacaine, given pre-operative, removed 48 h post surgery	Ultrasound guidance	No block IV morphine	Paracetamol Tramadol Oral morphine liquid	__	__

CAM: Confusion Assessment Method; IV PCA: intravenous patient-controlled analgesia; MMSE: Mini-Mental State Examination; DSM-IV: Diagnostic and Statistical Manual of Mental Disorders, Fourth Edition

**Table 3:** Demographics of included studies

Study	Design	Country	Age (mean ± standard deviation)	
			**Intervention**	**Control**
Nie 2015 [53]	Prospective, randomised controlled trial	China	73.6(2.1)	68.2(2.1)
Hao 2019 [42]	Double blind randomised controlled trial	China	72.3(3.78)	72.52(4.26)
Bielka 2021 [49]	Randomised controlled trial	Ukraine	71(3.7)	71.7(2.22)—spinal 73.0(1.5)—general
Yamamoto 2019 [43]	Prospective, two arm, parallel group, randomised controlled trial	Japan	84.7(6.5)	84.6(7.8)
Tang 2021 [44]	Prospective, controlled, and two parallel-group clinical trial	China	76.6(6.89)	78.0(6.45)
Mouzopoulos 2009 [50]	Randomised placebo-controlled trial	Greece	72.3(4.1)	73.1(3.8)
Morrison 2016 [51]	Multi-site randomised controlled trial	USA	83.4(8.49)	81.7(8.81)
Uysal 2020 [45]	Randomised controlled trial	Turkey	81.41(8.06)	82.04(6.83)
Cui 2024 [46]	Randomised controlled trial	China	72.6(3.9)	71.8(4.3)
Yoo 2024 [47]	Randomised controlled trial	South Korea	73.68(12.99)	70.87(13.42)
Loessin 2019 [48]	Randomised controlled trial	Canada	__	__
Rowlands 2018 [52]	Prospective randomised controlled pragmatic trial	UK	83.0(5.81)	83.9(6.24)

### Risk of bias assessment

**Fig. 2:**
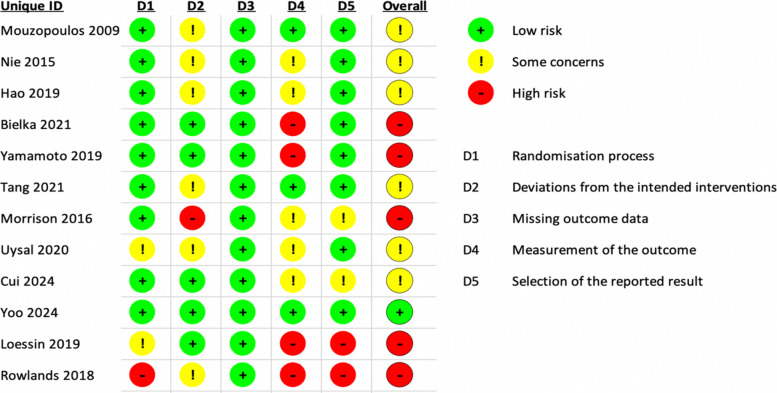
Risk of bias assessment

Overall, five studies [43, 48, 49, 51, 52] were assessed to be at high risk of bias, six studies [42, 44–46, 50, 53] had some concerns, and only one study [47] was deemed to be at low risk (Fig. 2). All but three studies were at low risk of bias due to the randomisation process [42, 43, 46, 47, 49–51, 53], with Uysal et al. and Loessin et al. having some concerns, and Rowlands et al. having a high risk. For bias due to deviations from the intended interventions, five studies [43, 46–49] were classified as low risk, six [42, 44, 45, 50, 52, 53] had some concerns, and one [51] was at high risk of bias. All studies were assessed to have a low risk of bias due to missing outcome data. Three studies [44, 47, 50] had a low risk of bias due to measurement of the outcome, five [42, 45, 46, 51, 53] had some concerns in this field, and four [43, 48, 49, 52] were classified as high risk. In the selection of the reported results, eight studies [42–45, 47, 49, 50, 53] were evaluated as low risk of bias, two [46, 51] had some concerns and two [48, 52] were reported as having a high risk.

### Meta-analysis

All but one study reported on the total incidence of POD. The study by Cui et al. reported on the presence of POD on each day following surgery for four days, but did not include a total incidence. To avoid overcounting, we selected data from day 2 for inclusion in our meta-analysis. In the study by Morrison et al., delirium incidence was presented as a percentage. Exact figures were obtained through contact with the author via email. The study by Bielka et al. involved two control groups with no nerve block, one with spinal anaesthesia, and one with general anaesthesia. For meta-analysis, data from both groups were combined for comparison with the intervention group.

**Fig. 3:**
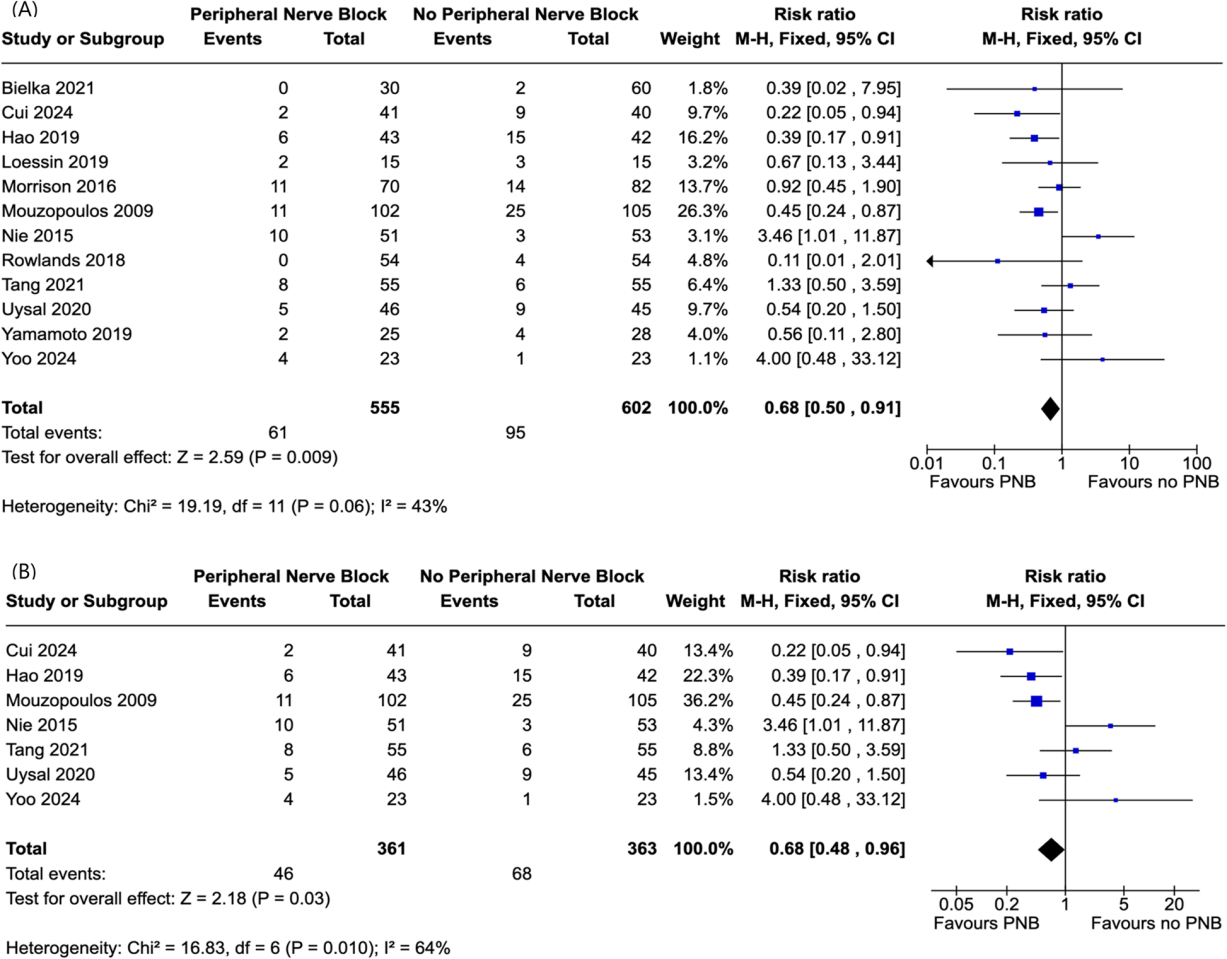
Forest plot of the effect sizes of peripheral nerve block on total incidence of post-operative delirium. **A** Forest plot with all eligible studies. **B** Sub-group analysis with studies with a high risk of bias excluded

This meta-analysis involved 12 studies with a total of 1157 participants, 602 in the control group and 555 in the intervention [42–53]. In the PNB group, the incidence of POD was lower than the control group (RR: 0.68, 95% CI [0.50 to 0.91], *P* = 0.009, I^2^ = 43%) (Fig. 3). In a sub-group analysis, with exclusion of studies with high risk of bias, the incidence of POD remained lower in the PNB group (RR: 0.68, 95% CI [0.48 to 0.96], *P* = 0.03, I^2^ = 64%) (Fig. 3).

### Level of certainty and Publication Bias

**Fig. 4:**
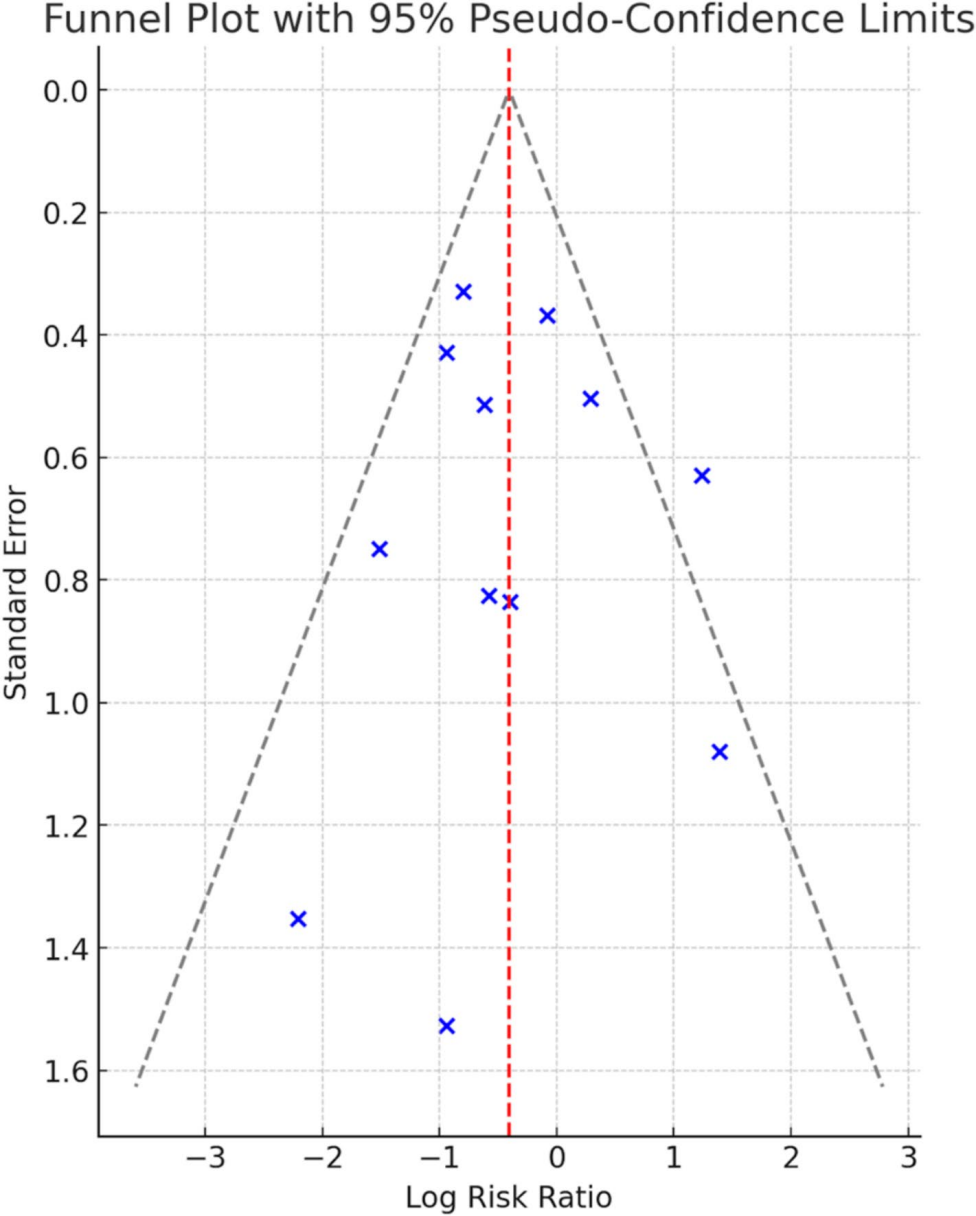
Funnel plot for publication bias of total incidence of post operative delirium

Since most of the studies had some concerns in the risk of bias assessment, but the effect was still present when high-risk studies were excluded in a subgroup analysis, the certainty of evidence was downgraded by one level due to risk of bias. Certainty of evidence was not downgraded for heterogeneity (I^2^ < 50%) or imprecision (*n* > 400). Regarding indirectness, the certainty of evidence was downgraded by one level as there were differences between studies in the interventions and comparators. The confidence interval (0.50–0.91) only included values of clinically meaningful effect, and the sample size was greater than 400, so certainty of evidence was not downgraded for imprecision. An Egger’s test was performed (*P* = 0.828), and no evidence of publication bias was identified as *P* > 0.05. Visual assessment of the funnel plot suggested potential asymmetry; however, the small number of studies made interpretation difficult (Fig. 4). Overall, the level of certainty was rated as moderate.

## Discussion

This systematic review and meta-analysis compared the effects of PNB versus no nerve block on POD in hip fracture patients. With an aging population in the UK, POD in older adults has gained attention due to its significant impact on surgical outcomes. Guidelines recommend the use of PNBs as analgesia in hip fractures, but there is a lack of strong evidence of their preventative effect on POD. This meta-analysis revealed that PNBs significantly reduced the incidence of POD.

According to the ICD-11 [11], delirium is defined as “An aetiologically nonspecific organic cerebral syndrome characterised by concurrent disturbances of consciousness and attention, perception, thinking, memory, psychomotor behaviour, emotion and the sleep–wake schedule” [12]. POD can manifest in a range of ways, with variability in duration, severity, and activity. There are three subtypes: hypoactive, hyperactive, and mixed. The characteristics are described in the NICE Clinical Guideline CG103 (Table 4) [13].

**Table 4:** Summary of the distinct presentations of the clinical subtypes of delirium

Subtype	Features
Hyperactive	Restlessness, agitation, aggression, sleep disturbance, delusions, hallucination, confusion
Hypoactive	Quietness, drowsiness, withdrawn, reduced appetite, movement, awareness, and concentration
Mixed	Fluctuation between hyperactive and hypoactive features

In the UK, postsurgical assessment of delirium uses the Confusion Assessment Method for the Intensive Care Unit (CAM-ICU) or the Intensive Care Delirium Screening Checklist (ICDSC) [14]. Due to variation in presentation, delirium is underdiagnosed. To avoid delays in treatment, health care practitioners must be trained to recognise features of POD [15].

The observed association may be explained by the ability of PNBs to reduce two primary precipitating factors for delirium: pain and systemic opioid use [54] Through reducing the need for opioid-based analgesics and limiting their sedating side effects, PNBs may mitigate the risk of delirium. Furthermore, PNBs offer sustained analgesia without relying on patient-initiated requests. Delirious patients are unable to appropriately recognise or communicate their pain scores and receive less PRN analgesia [55] A vicious cycle can be seen here, where unrelieved pain exacerbates delirium, which, in turn, impairs the patient’s ability to seek relief. Long-acting PNBs may help to interrupt this cycle and improve postoperative outcomes.

This systematic review displays several strengths. First, we achieved a comprehensive search strategy across multiple databases and reviewed a large cohort of studies, which reduced the potential for publication bias. We adhered to PRISMA 2020 guidelines and conducted a rigorous data screening and extraction process. Furthermore, we successfully received responses from study authors in the case of unclear data, which improved data accuracy and completion. Additionally, the included data was not limited to a single country or time period, further enhancing the generalisability of the findings.

Nevertheless, our findings must be interpreted considering key limitations. Considerable clinical heterogeneity was present across the studies. There were differences in the type, administration, and timing of the nerve blocks, as well as the additional surgical anaesthesia and supplementary analgesia. The PNBs were administered preoperatively, intraoperatively, and postoperatively in the form of single bolus injections, repeated injections, and catheter infusions. Rescue analgesia varied between studies, including but not limited to acetaminophen, NSAIDs, and morphine, in numerous modalities and doses. Only two studies [42, 50] used placebo or “sham” PNBs in the control group, reflecting the ethical implications of this technique. There were also differences in the assessment of delirium. Most studies used CAM, but one used the DRS-R-98, and two did not specify the tool used. The timing of delirium assessment was also inconsistent; this information can be viewed in Table 2. While some studies maintained the same surgical anaesthesia technique across both the control and intervention groups, two studies [51, 52] included a mix of techniques, with only Rowlands et al. providing commentary on the comparability between groups. Despite these variations, heterogeneity was statistically low to moderate, and our robust meta-analysis continued to demonstrate a significant effect. This highlights the clinical importance of PNBs in reducing POD, irrespective of diversity in study design or block techniques.

Data on other relevant risk factors, such as anaemia and dehydration, were insufficient for subgroup analysis and are acknowledged as limitations of this study. Another significant limitation of this review is the exclusion of studies that included patients who were delirious or cognitively impaired pre-operatively. We found that these studies did not stratify outcomes by cognitive status and that a potential benefit of PNBs in cognitively impaired patients may be diluted in the overall analysis of aggregated data. It was decided that this led to an underestimation of the true effect and limited the interpretability; thus, these studies were excluded. Notably, a meta-analysis by Kim et al. [56] included randomised controlled trials (RCTs) with mixed populations of patients both with and without cognitive impairment, and conducted a subgroup analysis of these trials. The meta-analysis by Bin et al. [57] also included studies with cognitively impaired patients. Ultimately, both papers concluded that PNBs do not affect POD in this population, which seems unlikely. We recommend that future RCTs investigate the impact of PNBs on POD within clearly defined subgroups based on preoperative cognitive status. Preoperative cognitive impairment represents a heterogeneous spectrum in the elderly: some patients have longstanding diagnoses such as dementia, others exhibit transient pre-operative delirium in the context of acute illness, and some remain cognitively intact preoperatively. Stratification of future outcomes according to distinct cognitive profiles—using validated assessment tools in conjunction with collateral history and medical records—may provide greater clarity on the effectiveness of PNBs across groups of varying cognitive status.

This review is an update of the existing work [40, 56, 57]. It builds on the findings with the addition of more recent RTCs, application of a stricter quality assessment, and a subgroup analysis with exclusion of studies at high risk of bias.

It is important to acknowledge the barriers to PNBs placement and address the “knowledge to action gap” to improve clinical outcomes in hip fractures. Implications include stretched human resources, theatre time pressures, lack of education, and a lack of protocols or workflow processes [58]. To address these pressures, ultrasound-guided nerve block training should be provided to anaesthesiologists and doctors in the emergency department for early block placement. There is scope for the training of advanced clinical practitioners in this skill to supplement the workforce. A designated area and equipment for the administration of nerve blocks should be considered to improve consistency of care and maintenance of skill [59]. It can be argued that funding these resources will provide an overall economic benefit to trusts by reducing complications and the length of hospital stays in elderly hip fractures.

While these resources will require funding, reductions in complications, hospital length of stay, and overall healthcare costs in elderly hip fracture patients may result in a net economic benefit for trusts.

## Conclusion

In summary, this meta-analysis supports the use of PNBs as a strategy to reduce POD in cognitively intact adults undergoing hip fracture surgery. However, evidence remains limited regarding their effectiveness in cognitively impaired populations. Future research should aim to stratify patients by cognitive status and evaluate PNBs as part of an integrated, multimodal strategy for POD prevention.

## Data Availability

Contact the authors for access to any additional data and materials.

## Abbreviations

PNB: Peripheral nerve block
POD: Post operative delirium
CAM: Confusion Assessment Method
IV PCA: Intravenous patient-controlled analgesia
MMSE: Mini-Mental State Examination
DSM-IV: Diagnostic and Statistical Manual of Mental Disorders, Fourth Edition
RTC: Randomised controlled trial
